# BmCPV-Derived Circular DNA vcDNA-S7 Mediated by *Bombyx mori* Reverse Transcriptase (RT) Regulates BmCPV Infection

**DOI:** 10.3389/fimmu.2022.861007

**Published:** 2022-03-15

**Authors:** Min Zhu, Jun Pan, Xinyu Tong, Qunnan Qiu, Xing Zhang, Yaxin Zhang, Sufei Sun, Yongjie Feng, Renyu Xue, Guangli Cao, Xiaolong Hu, Chengliang Gong

**Affiliations:** ^1^ School of Biology and Basic Medical Science, Soochow University, Suzhou, China; ^2^ Institute of Agricultural Biotechnology and Ecological Research, Soochow University, Suzhou, China

**Keywords:** BmCPV, viral circular DNA, vcDNA-S7, reverse transcriptase activity, RNAi

## Abstract

Circular DNAs derived from single-stranded RNA viruses play important roles in counteracting viral infection. However, whether double-stranded RNA viruses generate functional circular DNAs is still unknown. Using circDNA sequencing, divergent PCR, DNA *in situ* hybridization and rolling circular amplification, we presently confirmed that in silkworm, *Bombyx mori* cytoplasmic polyhedrosis virus (BmCPV), a double-stranded RNA virus belonging to cypovirus, is prone to produce a BmCPV-derived circular DNA termed as vcDNA-S7. We have also found that vcDNA-S7 formation is mediated by endogenous reverse transcriptase (RT), and the proliferation of BmCPV can be inhibited by vcDNA-S7 *in vitro* and *in vivo*. Moreover, we have discovered that the silkworm RNAi immune pathway is activated by vcDNA-S7, while viral small interfering RNAs (vsiRNAs) derived from transcribed RNA by vcDNA-S7 can be detected by small RNA deep sequencing. These results suggest that BmCPV-derived vcDNA-S7, mediated by RT, can serve as a template for the biogenesis of antiviral siRNAs, which may lead to the repression of BmCPV infection. To our knowledge, this is the first demonstration that a circular DNA, produced by double stranded RNA viruses, is capable of regulating virus infection.

## Introduction

Due to its limited genome size and more restricted number of genes, viruses typically depend on host cells to achieve its proper infection and proliferation. Thus, viruses mostly fulfill their own life cycle, involving processes such as replication, transcription, and virus assembly, by hijacking or utilizing host factors. At the same time, the host starts its own defense system to resist the infection of the virus, as soon as virus invasion is surveilled (1–3). Therefore, both host and virus act as “opponents” fighting for their own survival.

Circular DNAs (circDNAs) are ubiquitous forms of DNA that are generally found in the mitochondria and chloroplasts of eukaryotes, bacteria, and DNA viruses. Recent studies have reported that chromosomes from a variety of eukaryotes can form extrachromosomal circular DNAs (eccDNAs) that may play important roles during tumorigenesis (4–7). In addition, some studies have shown that certain single-stranded RNA viruses can produce viral circular DNAs (vcircDNAs), mediated by the host retrotransposon, which have an important role in controlling virus infection (8, 9). For instance, the genomic RNA of a number of viruses, such as *Drosophila* C virus (DCV), flow house virus (FHV), Sindbis virus (SNV), and chikungunya virus (CHIKV), can form stable and chimeric vcircDNAs, mediated by retrotransposons. These vcircDNAs have transcriptional ability and can stably (and continuously) transcribe RNAs to produce a higher abundance of vsiRNAs, leading to a persistence of the viral infection by RNAi pathways (8, 9). However, all reported vcircDNAs are circDNAs derived from single-stranded RNA viruses. In fact, it is not yet known whether double stranded RNA viruses can also form vcircDNAs during viral infection.

*Bombyx mori* cytoplasmic polyhedrosis virus (BmCPV) is a typical species of cytoplasmic polyhedrosis virus (Reoviridae family), whose genome contains 10 double-stranded RNA segments. BmCPV specifically infects the midgut cells of silkworm, leading to the occurrence of cytoplasmic polyhedrosis (10) and consequent failure of sericulture. It has been reported that BmCPV can enter cells *via* the endocytic pathway (11, 12) and evade the silkworm immune-related pathways, such as Toll and IMD (13), which hijacks silkworm mRNAs (14–17), lncRNAs (18), miRNAs (19), circRNAs (20), protein expression (21) as well as metabolic progression (22) to favor the proliferative replication of viruses. In addition, studies have indicated that the BmCPV-related transcripts can be processed to form viral miRNAs and promote viral infection and favors viral replication (23, 24). However, how does silkworm may defend itself from BmCPV invasion? Studies have indicated that, when attacked by BmCPV, the defense system of silkworm can target the BmCPV genome as well as its transcripts to produce virus-derived small interfering RNAs (vsiRNAs) (25) as a means to resist virus infection. A recent study has shown that silkworm can also use BmCPV S5 segment dsRNA to encode small peptide vSP27 and control BmCPV infection (26, 27). Altogether, these results deepen the understanding of BmCPV interaction with silkworm and the control of BmCPV infection process. Yet, we believe that additional BmCPV-derived molecules may also be produced to control the virus infection process.

In this study, we confirmed that in silkworm, BmCPV can produce a circDNA termed as vcDNA-S7 whose formation is mediated by reverse transcriptase (RT) and the proliferation of BmCPV can be inhibited by vcDNA-S7. Moreover, we found that the RNAi pathway can be activated by vcDNA-S7, while viral small RNAs derived from transcribed RNA by vcDNA-S7 can be detected by small RNA deep sequencing. These results suggest that the BmCPV-derived vcDNA-S7 mediated by reverse transcriptase can regulate BmCPV infection *via* RNAi pathway. Collectively, our results show that silkworm can restrict BmCPV infection by a novel nucleic acid-based antiviral mechanism, which is dependent on RT activities.

## Materials and Methods

### BmCPV Virions Preparation

BmCPV virions was prepared according to our previous reports (12). Briefly, the 3rd instar silkworms were fed with fresh mulberry leaves, coated with 10^8^/ml of BmCPV polyhedra suspension, for 8 h. After further feeding, midgut of infected silkworms was dissected at 7–10 days post-infection. Polyhedra was purified from midgut. To obtain related virions, purified polyhedra was resuspended with 0.01 mol/L Na_2_CO_3_-0.09 mol/L NaHCO_3_ buffer at 28°C for 20 min, followed by adjustment of pH to 8.0 with 1 M HCl. BmCPV virions were stored at 4°C for subsequent studies.

### BmCPV Infection

The BmN cell line was derived from silkworm ovary. Previously, we have developed an BmCPV *in vitro* infection model based on BmN cells to study BmCPV–silkworm interaction in an easier-to-handle *in vitro* system (12, 27–29). Herein, the cells (1×10^5^/well) were seeded in 6-well plates and cultured for 48 h in TC-100 insect medium (Germany, PanReac Applichem, A2017) supplemented with 10% fetal bovine serum (USA, BI, 04-001-1A-US). Then, the medium was removed and retained, and cells were incubated for 1 h with 5 µL BmCPV virions (1×10^5^ cleaved polyhedra/µL) in serum free TC-100 medium. After that, cells were carefully washed once with phosphate-buffered saline (PBS) and cultured in complete medium. Fourth instar silkworms (strain Jingsong) were injected with 1µL BmCPV virions (1×10^5^ cleaved polyhedra/µL).

### Circle-Seq

The midgut of 4th instar BmCPV-infected silkworms (Jingsong strain) was dissected and circDNA sequencing was performed by CloudSeq Biotech Inc. (Shanghai, China) according to published procedures with slight modification ^[1]^. Briefly, high molecular weight DNA was extracted from the midgut of infected silkworm with BmCPV, followed by removing linearized DNA with exonucleases Plasmid-Safe ATP-dependent DNase (USA, Epicentre, E3101K) to obtain eccDNAs. The enriched eccDNAs were amplified by phi29 polymerase (China, Beyotime, D7053S). This amplified DNA was sheared and fragmented by sonication (Belgium, Bioruptor) and then utilized for the library construction using the NEBNext^®^ Ultra II DNA Library Prep Kit for Illumina (USA, New England Biolabs). Sequencing procedures were carried out according to the manufacturer’s instructions (USA, Illumina NovaSeq 6000 with 150bp paired-end reads). For Circle-seq data analysis, Fastqc software was used to evaluate the quality of the original data (30). This analysis was followed by a comparison between the original data and the BmCPV genomic RNA sequences using BWA software. The processing of the sam file to fit the format required by Circle-MAP was performed using Sam tools (30). Finally, eccDNA was identified by Circle-MAP, and respective genes were annotated accordingly (30). The raw sequencing data have been uploaded to NCBI database (accession number SRR16977180).

### DNA Extraction and vcDNA-S7 Test

BmCPV-infected silkworm midgut or cells were grounded with cold 1×PBS. Thereafter, DNA was purified by phenol-chloroform extraction. The isolated DNA samples were incubated with RNase A (USA, Thermo Fisher, Cat: EN0531) and plasmid-safe DNase (USA, Epicentre, Cat: E3101K) mix at 37°C for 30 min. Reactions were terminated by addition of 10 mM EDTA. PCR was then performed using mock-treated or DNase-digested samples. Primer sequences used for the identification of viral circDNA (vcDNA-S7), derived from BmCPV genomic RNA S7, are shown in **Table S1**.

### DNA FISH

DNA FISH assays were performed using a Ribo™ Fluorescent *in situ* Hybridization Kit (China, BOSTER, Cat: MK1030) according to the manufacturer’s protocol. The FISH probe targeting the junction site sequence of vcDNA-S7 (5’Biotin-CAGACGCCAACAAGGATCCTCAACCAC) was synthesized by Sangon Biotech (China, Shanghai). In brief, 1×10^4^ BmN cells were digested with proteinase K at 48 h post-infection of BmCPV. Then, BmCPV-infected BmN cells were hybridized with FISH probe at 37°C overnight. Hybridization signals were detected using CY3 labeled streptomycin. Cell nuclei were counterstained with 4, 6-diamidino-2-phenylindole (DAPI). Cell images were captured using a Leica DM2000 microscope (Leica, Wetzlar, Germany).

### Rolling Circular Amplification (RCA)

RCA can be used to confirm the circular configuration of a targeted molecule (31). For this, the total DNA of silkworm midgut infected with BmCPV was extracted. After linear DNA and RNA were respectively removed using DNase- plasmid safe and RNase A, RCA was carried out with a vcDNA-S7-F primer (which crossed the junction site of vcDNA-S7) in a final reaction volume of 20 µl containing 1×buffer, 1×dNTP mix, 1μg DNA, 2.5 µM primer, and ddH_2_O (up to 19 µl). In this experiment, the sequence flanking the junction site of vcDNA-S7 was cloned into the vector pMD-19T (China, TaKaRa, D104A) to construct the plasmid pMD19T-S7 as positive control. The RCA reaction was incubated at 95°C for 5 min and rapidly placed in an ice bath for 2 min, followed by the addition of 1 µl (10 U) phi29 DNA polymerase (China, Beyotime, Cat: D7053S) and then incubated at 30°C for additional 2 h. After a final incubation at 65°C for 10 mins, the RCA products were identified by PCR using primers (vcDNA-S7F and vcDNA-S7R) (**Table S1**).

### Drug Treatment

Azidothymidine (AZT) is a widely used RT inhibitor that can inhibit endogenous RT activity (9). GSK-LSD1, an inhibitor of histone H3 lysine 9 (or histone H3 lysine 4) demethylases, has been reported to increase the methylation level of histone H3 lysine 9, thus increasing RT activity in mammalian cells. In this study, AZT (China, Absin, Cat: abs810874) and GSK-LSD1 (USA, Selleck, Cat: S7574) were used to modulate the retrotransposon activity of RT in BmN cells. MTT assays were carried out to determine the proper drug concentration for subsequent experiments. Briefly, 10^3^ BmN cells were seeded in 96-well plates and treated with different concentrations of AZT (0, 1, 5, 10, 25, 50, 100 mM) or GSK-LSD1 (0, 1, 5, 10, 25, 50, 100 nm) for 24 h. Cells viability was evaluated by MTT. According to MTT assay, BmN cells were treated with 5 mM AZT and/or 50 nM GSK-LSD1 in the subsequent experiments. Before further treatments, BmN cells were seeded in 6-well plates at the density of 1× 10^6^ cells/well and cultured for one day. Thereafter, cells were pretreated with 5 mM AZT for 4–6 h or 50 nM GSK-LSD1 for 24 h, followed by BmCPV infection and other downstream assays.

### Sequence Alignment Analysis of the Silkworm RT

To determine the source of RT activity involved in the formation of vcDNA-S7, sequence alignment analysis of the silkworm RT was carried out using NCBI Neighbor Joining Blast (https://www.ncbi.nlm.nih.gov/blast/treeview/treeView.cgi?request=page&blastRID=YEZ2B06S016&queryID=gb|AAA17752.1|&entrezLim=&ex=&exl=&exh=&ns=100&screenWidth=1920&screenHeight=1080). The RT sharing homologous amino acid sequences were divided into a group.

### siRNA Knockdown

Gene-specific siRNAs were designed and synthesized by Sangon Biotech (Shanghai, China). The sequences of respective siRNAs targeting RT genes are shown in **Table S2**. A total of 100 pmol siRNA was transfected into 1×10^6^ BmN cells using Roch-X gem (Switzerland, Roche, Cat: 6366236001). At 48 h post-transfection, cells were infected with BmCPV, followed by real-time PCR to evaluate the expression level of target genes as well as the number of vcDNA-S7 copies at 48h post-infection.

### Reverse Transcriptase (RT) Activity Assay

RT activity assay was performed according to previous studies (32). For this, BmN cells were washed three times with cold PBS and then lysed with CHAPS lysis buffer (China, ZYSW, ZY-25-01380) supplemented with 1×protease inhibitor (Switzerland, Roche, 11873580001). Cell lysis was performed at 0°C for 30 min, followed by centrifugation at 13,000 rpm at 4°C for 10 min to collect respective supernatants. BCA Protein Assay Kit (China, Sangon, C503021) was used to determine the protein concentration of each cell extract. RT activity was tested as follows. *In vitro* transcribed green fluorescent protein (GFP) gene RNA was served as a template in a 20 μl reaction volume, containing 10 μg or 1 μg of cell extracts, 10 ng of GFP RNA, 20 U of RNase inhibitor (USA, Promega, N251B), 10 nmol of GFP reverse primer, 0.2 mM dNTP, 1× reverse transcription buffer (China, TransGen, AT101) and nuclease-free water. The reaction mixture was incubated at 25°C for 5 min, and at 37°C for 30 mins, followed by incubation at 85°C for 5 min. Reactions devoid of cellular protein extract were set as negative controls. One unit of M-MLV reverse transcriptase (China, TransGen, AT101) was used as a positive control. Reverse-transcribed GFP cDNA was quantitated by real-time PCR using GFP-specific primers (**Table S1**). The relative RT activities between samples were compared according to the GFP cDNA levels.

### Preparing vcDNA-S7 *In Vitro*

Based on the analysis of vcDNA-S7 linear sequence, there was a *Nhe*I restriction endonuclease site at its 3’ terminal end. Therefore, we designed specific primers carrying a *Nhe*I site and then performed PCR using a plasmid pIZT-CS7 containing the full-length cDNA of BmCPV genomic dsRNA S7 segment as a template. The amplified product was further digested with *Nhe*I and then circularized to obtain vcDNA-S7, using T4 DNA ligase. Finally, the ligation products were treated with plasmid safe™ ATP-dependent DNase in order to remove any linear and non-circular DNA. A flowchart for the *in vitro* synthesis of vcDNA-S7 is presented (**Figure S1**).

### Quantitative Real-Time PCR

To explore the copy numbers of vcDNA-S7, 1×10^6^ BmN cells were firstly inoculated with BmCPV. DNA was further extracted from infected cells, and the copies of vcDNA-S7 were determined by absolute quantitative real-time PCR using primers vcDNA-S7F and vcDNA-S7R (**Table S1**). Total RNA was extracted from BmN cells or silkworm tissues, and then reverse transcribed to cDNA using random primers (First Strand cDNA Synthesis Kit, Transgene, Beijing, China). The expression levels of genes were determined by real-time PCR using primers as shown in **Table S1**. The expression level of translation initiation factor eIF-4A *(TIF-4A)* gene was utilized as normalizer.

### Western Blotting

Total protein lysates (30 µg/lane) were separated by SDS-PAGE and transferred onto Nitrocellulose filter membrane (USA, Millipore, HATF00010). Membranes were blocked with 3% BSA in phosphate-buffered saline (PBS) containing 0.05% Tween 20, followed by incubation with respective primary antibodies. The primary antibodies used were anti-H3K9me3 (China, ABclonal, A2360), anti-α-tubulin (USA, Proteintech, 66031-1-Ig), anti-BmCPV VP7 (viral structural protein 7) (27, 29) and anti-BmCPV Polh (polyhedrin) (prepared by our laboratory). The secondary antibodies presently used were HRP-labeled goat anti-rabbit IgG or goat anti-mouse IgG, at 1:5000 dilution (USA, Proteintech, SA00001-1). Protein-specific signals were measured using an enhanced chemiluminescence (ECL) Western blot detection kit (Sangon, Shanghai). Quantitative analysis of the visible bands was performed by ImageJ program.

### Small RNA Sequencing

To assess whether small RNAs derived from vcDNA-S7 were present *in vitro*, 1×10^7^ BmN cells were transfected with 6 µg vcDNA-S7. The BmN cells were collected at 48 h post-transfection, and small RNAs was then extracted for deep sequencing. Sequencing was carried out by Shanghai oebiotech Co., Ltd (Shanghai, China). The obtained small RNAs were further analyzed using vcDNA-S7 as a reference sequence. The raw sequencing data have been uploaded to NCBI database with the accession number SRR17050376.

### Statistical Analyses

Data was expressed as mean ± SD (standard deviation). Statistical analyses were performed by one-way ANOVA and t-test to determine statistical significance between the groups, using GraphPad Prism6 software. *p*-value ≤0.05 was considered as a threshold of statistical significance.

## Results

### BmCPV-Derived vcDNA-S7 Is Present in BmCPV-Infected Cells

**Figure 1:**
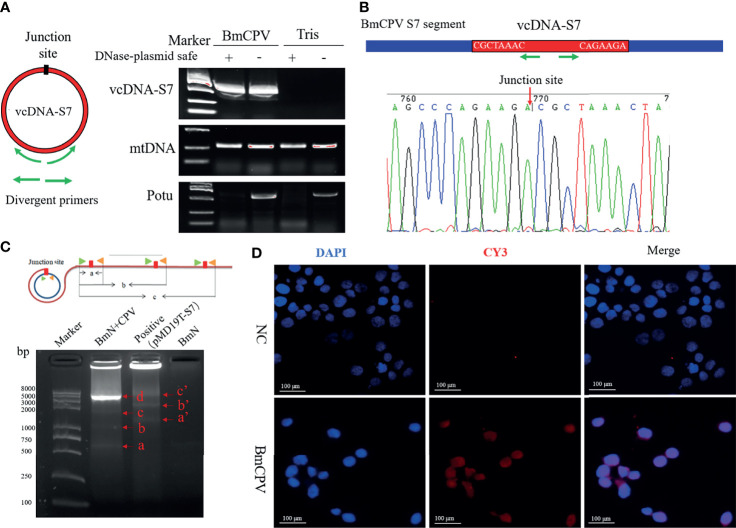
*mori* produces BmCPV-derived circular DNA vcDNA-S7. **(A)** vcDNA-S7 was verified by divergent PCR. left, Schematic of primer to validate the junction site of vcDNA-S7. Green arrow represents divergent primers. Right, electrophoresis of PCR product from flanking sequence of the junction site of vcDNA-S7. DNA from BmCPV infected silkworm midgut and healthy silkworm midgut were extracted. RNAs and linear DNAs were respectively removed with RNase A and Plasmid-Safe™ ATP-Dependent DNase. mtDNA representing mitochondrial DNA as a positive control; Potu representing genomic linear DNA as a negative control. **(B)** Sanger sequencing of the products of divergent PCR. The green arrow represents the divergent primer and the red arrow represents the junction site of vcDNA-S7. **(C)** RCA validated that vcDNA-S7 is a circular molecule. Upper, schematic of RCA of circular molecules. Under, electrophoretic detection of RCA products. ‘BmN+CPV’ represents the BmN cells infected with BmCPV. The plasmid pMD19T-S7 containing the sequence flanking the junction site of vcDNA-S7 used as positive control. The genomic DNA from BmN cells were used a negative control. **(D)** Validation of vcDNA-S7 by *in situ* hybridization. Top row is normal cultured BmN cells and bottom row is BmN cells infected with BmCPV. Red fluorescence indicates the biotin labeled vcDNA-S7-specific probe, and blue indicates that the nuclei were stained by DAPI.

circDNAs ubiquitous forms of DNA that are generally found in DNA viruses, bacteria, as well as the mitochondria and chloroplasts of eukaryotes. Recent studies have reported that chromosomes of a variety of eukaryotes can form eccDNAs to perform important biological functions (4–7). To explore whether BmCPV infection could affect the generation of eccDNAs in *B. mori*, we performed circDNA sequencing of midgut from *B. mori* infected with BmCPV. Previous studies have shown that some single-strand RNA viruses are able to form viral circDNAs (vcircDNAs) (9). Interestingly, some BmCPV-derived vcircDNAs have also been identified when we presently aligned the sequenced data with BmCPV genome. Herein, we selected a vcircDNA termed as vcDNA-S7 with the highest abundance from BmCPV genomic dsRNA S7 for further investigations. The vcDNA-S7 is derived from the region 426-1045 nt of the sense strand of BmCPV genomic dsRNA S7 (GenBank: GQ150538.1). To further confirm whether vcDNA-S7 is a circular DNA molecule, total DNA was extracted from silkworm midgut infected with BmCPV. The obtained DNA was analyzed by PCR using primer pairs for the respective detection of vcDNA-S7s, mitochondrial DNA and the promoter of *Bmotu* gene (Potu) after treatment with DNase Plasmid-Safe to properly remove linear DNA. Specific PCR products amplified from mitochondrial (mtDNA) and Potu DNAs were observed in samples not treated with DNase Plasmid-Safe (**Figure 1A**). However, only mtDNA was amplified in samples treated with DNase Plasmid-Safe, suggesting that linearized DNA was selectively removed by this DNase treatment (**Figure 1A**). vcDNA-S7 was present in both DNase-treated and non-treated DNA samples from BmCPV-infected silkworm, but not in uninfected silkworm. Furthermore, the junction site representing vcDNA-S7 was confirmed by Sanger sequencing of PCR product (**Figure 1B**). A RCA assay (31) was further performed to confirm whether vcDNA-S7 structure is circular. As shown in **Figure 1C**, we observed RCA products at different molecular weights that were consistent with the theoretical values (**Figure 1C**). Moreover, *in situ* hybridization targeting vcDNA-S7 was conducted in BmN cells infected with BmCPV. As expected, vcDNA-S7 was identified in BmCPV-infected cells (**Figure 1D**). Collectively, our results confirmed that vcDNA-S7 is detectable in BmCPV-infected cells.

### RT Activity Affects the Formation of vcDNA-S7

**Figure 2:**
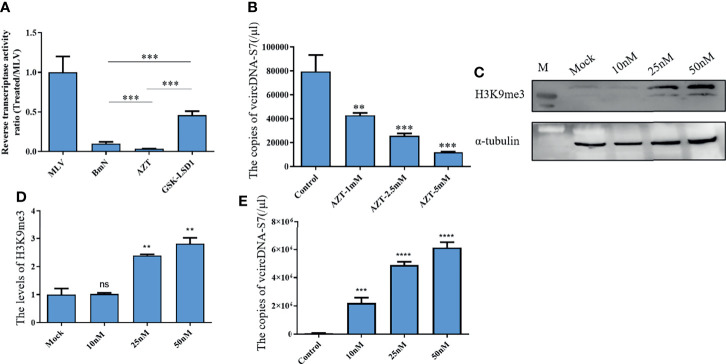
Reverse transcriptase activity is involved in the formation of vcDNA-S7. **(A)** Endogenous reverse transcriptase activity was measured in BmN cells. BmN cells were pre-treated with 5mM AZT or 50nM GSK-LSD1 for 24 h and then the cells were lysed and the reverse transcriptase activity was measured. Relative reverse transcriptase activity was calculated to 1U of commercialized M-MLV reverse transcriptase. **(B)** Effect of AZT treatment on the formation of vcDNA-S7. 1×10^6^ BmN cells were pre-treated with AZT at the indicated concentrations for 24 h and were infected with BmCPV for additional 48 h. The copies of vcDNA-S7 were determined by real-time PCR. **(C, D)** GSK-LSD1 treatment improved the level of histone H3K9me. 1×10^6^ BmN cells were pre-treated with GSK-LSD1 at the indicated concentrations for 24 h. The level of histone H3K9me was analyzed by western blotting **(C)**. The grayscale of Western blotting signal bands is analyzed by software Image J **(D, E)** Effect of GSK-LSD1 treatment on the formation of vcDNA-S7. 1×10^6^ BmN cells were pre-treated with GSK-LSD1 at the indicated concentrations for 24 h and were infected with BmCPV for another 48 h. The copies of vcDNA-S7 were determined by real-time PCR. (n=4, **P<0.01; ***P<0.001; ****P<0.0001; ns, no significance).

Viral RNAs can solely form DNA by reverse transcription. Specifically, retroviral RNAs can generate viral DNA (vDNA) by a RT encoded by the virus itself, while some non-retroviral RNAs can form vDNA using the RT of host retrotransposon (8). BmCPV is a non-retroviral RNA virus. As such, we speculated that the formation of vcDNA-S7 may also depend on silkworm RT activity. Previously, we have developed an BmCPV *in vitro* infection model utilizing BmN cells. Therefore, the effects of RT activity on the vcDNA-S7 formation were evaluated in BmN cells treated with RT inhibitor AZT or RT activator GSK-LSD1. As expected, AZT treatment was able to reduce RT activity (**Figure 2A**) as well as to inhibit vcDNA-S7 formation (**Figure 2B**). GSK-LSD1 treatment dramatically increased the level of H3K9me3 (**Figures 2C, D**) in BmN cells, as well as RT activity (**Figure 2A**). In particular, the formation of vcDNA-S7 was also promoted by GSK-LSD1 treatment (**Figure 2E**). Collectively, these results suggested that silkworm RT activity is involved in vcDNA-S7 formation.

**Figure 3:**
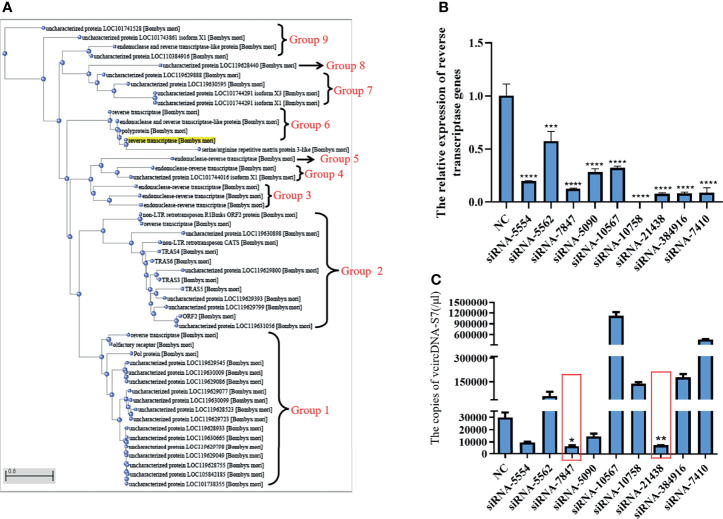
The source of reverse transcriptase activity involved in vcDNA-S7 formation. **(A)** Cluster analysis of genes encoding reverse transcriptase in silkworm. reverse transcriptases genes in the silkworm were divided into 9 groups. **(B)** Silencing efficiency of siRNAs on 9 groups of genes encoding reverse transcriptase. siRNA. 1×10^6^ BmN cells were transfected with siRNAs for 48 h, and were infected with BmCPV for another 48 h. Total RNA was extracted and the expression level of reverse transcriptase genes relative to housekeeper gene TIF-4A was detected by real-time PCR. **(C)** 1×10^6^ BmN cells were transfected with siRNAs for 48 h, and were infected with BmCPV for another 48 h. The copies of vcDNA-S7 were determined by real-time PCR. (n=3, *, *P*<0.05; **P<0.01; ***P<0.001; ****P<0.0001).

In order to determine the source of RT activity involved in the formation of vcDNA-S7, we carried out a sequence alignment analysis of the silkworm’s RT. As shown in **Figure 3A**, the RT of silkworm can be divided into 9 groups. We designed siRNAs based on the common sequence to knock down the expression of the corresponding RT genes. Analysis of siRNA interference efficiency showed that the designed siRNAs were able to inhibit the expression of corresponding RT genes (**Figure 3B**). When the RT genes in group 2 and 6 were silenced, the formation of vcDNA-S7 was inhibited (**Figure 3C**), suggesting that RT genes in group 2 and 6 were related to vcDNA-S7 formation. Since 18 RT genes are distinctively present in these two groups, the specific gene(s) involved in the formation of vcDNA-S7 is not fully clear. In the future, we expect to design specific siRNAs for each one of these 18 genes to better dissect which gene(s) is involved in vcDNA-S7 formation.

### vcDNA-S7 Inhibits the Proliferation of BmCPV

**Figure 4:**
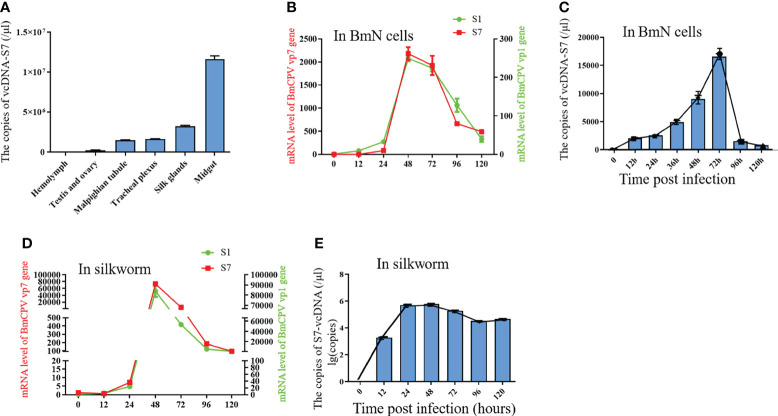
The expression pattern of vcDNA-S7. **(A)** The express pattern of vcDNA-S7 in different tissues of BmCPV infected silkworm. **(B)** mRNA levels of BmCPV vp1 and vp7 genes in BmCPV infected BmN cells at different stages were determined by real-time PCR. **(C)** copies of vcDNA-S7 in BmCPV infected BmN cells at different stages were determined by real-time PCR. **(D)** mRNA levels of BmCPV vp1 and vp7 genes in BmCPV infected silkworm midgut at different stages were determined by real-time PCR. **(E)** copies of vcDNA-S7 in BmCPV infected silkworm midgut at different stages were determined by real-time PCR (n=3).

To determine the biological functions of vcDNA-S7, we firstly explored the expression profile of vcDNA-S7 in different tissues of BmCPV-infected silkworm at the third day of the fifth instar. These tissues presently analyzed included the midgut, hemolymph, Malpighian tubule, ovary and testis, trachea plexus, and silk gland. Significant differences in vcDNA-S7 expression in different tissues were noticed, where the highest expression was observed in the midgut (**Figure 4A**). Since BmCPV can specifically infect the intestinal epithelial cells of silkworms, we suggested that vcDNA-S7 levels correlated with BmCPV infection. To understand the expression pattern of vcDNA-S7, the expression levels of vcDNA-S7 and viral structural protein 1 and 7 (VP1 and VP7) genes were determined by real-time PCR. In BmN cells, the expression of vp1 and vp7 genes increased rapidly and reached the peak at 48 h post infection (hpi), followed by a downward trend (**Figure 4B**). The expression pattern of vcDNA-S7 was similar to those of these viral genes (**Figure 4C**). Similar results were observed in the midgut of BmCPV-infected silkworm (**Figures 4D, E**).

**Figure 5:**
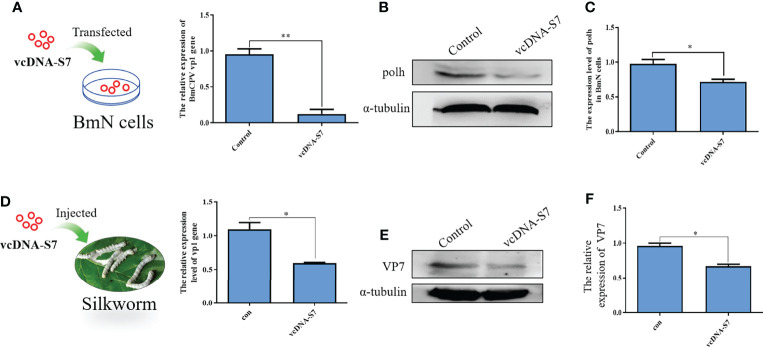
vcDNA-S7 inhibited BmCPV infection. **(A–C)** vcDNA-S7 down-regulated the expression level of BmCPV vp1 gene and polh protein in BmN cells. BmN cells (1×10^6^) were transfected with 1 μg vcDNA-S7 for 24 h and were infected with BmCPV for another 48 h. The expression levels of vp1 gene relative to translation initiation factor (TIF-4A) were investigated by real-time PCR **(A)**. The level of Polh protein was analyzed by western blotting **(B)**. The grayscale of western blotting signal bands is analyzed by software Image J **(C)**. **(D–F)** Injection of vcDNA-S7 into silkworm down-regulated the expression level of BmCPV vp1 gene and VP7 protein in BmN cells. The silkworm larvae of fourth instar were injected 1μg of vcDNA-S7 for 24 h and were infected with BmCPV for another 48 h. The expression levels of vp1 gene relative to translation initiation factor (TIF-4A) were investigated by real-time PCR **(D)**. The protein level of VP7 was analyzed by western blotting **(E)**. The grayscale of western blotting signal bands is analyzed by software Image J **(F)**. (n=2, *P<0.05; **P<0.01).

Previous studies have indicated that RNA virus-derived DNAs may play roles in antiviral response of host (8, 9). To test the function of vcDNA-S7, BmN cells were transfected with vcDNA-S7, followed by infection with BmCPV at 24h post transfection. Total RNA and protein were extracted at 48 hpi, and the relative expression levels of BmCPV vp1 gene and Polh protein were detected by real-time PCR and Western blotting, respectively. As shown in **Figures 5A–C**, the levels of vp1 gene and Polh protein decreased in transfected cells with vcDNA-S7. To validate these results *in vivo*, 1 µg vcDNA-S7 was injected into the larvae at day 3 of fourth instar. At 24 h post-injection, silkworms were infected with BmCPV, and total RNA and protein of silkworm midgut were extracted at 48 hpi to verify the relative expression levels of BmCPV vp1 gene as well as VP7 protein. Our results indicated that, the expression levels of BmCPV vp1 gene as well as VP7 protein decreased in the midgut of injected silkworm with vcDNA-S7 compared with the control (**Figures 5D–F**). These data suggested that vcDNA-S7 can inhibit BmCPV infection.

**Figure 6:**
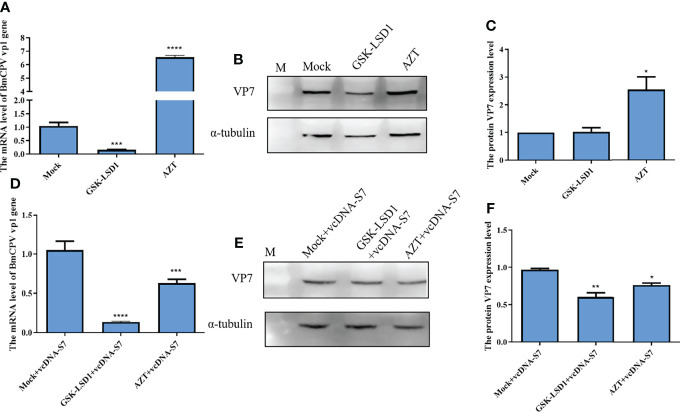
Endogenous reverse transcriptase activity inhibited BmCPV infection. **(A–C)** Endogenous reverse transcriptase activity inhibited virus infection in BmN cells. 1×10^6^ BmN cells were pre-treated with 5 mM AZT or 50 nM GSKLSD1 and infected with BmCPV for 24 h. The relative RNA level of BmCPV vp1gene was determined by real-time PCR **(A)**. The protein level of VP7 was analyzed by western blotting **(B)**. The grayscale of western blotting signal bands is analyzed by software Image J **(C)**. **(D–F)** Effect of endogenous reverse transcriptase activity on the inhibitory effect of vcDNA-S7 on virus. 1×10^6^ BmN cells were pre-treated with 5mM AZT or 50 nM GSKLSD1 and were transfected 1μg vcDNA-S7 for 24 h. Then, the cells were infected with BmCPV for another 48 h. The relative RNA level of BmCPV vp1gene was determined by real-time PCR **(D)**. The level of VP7 protein was analyzed by western blotting **(E)**. The grayscale of western blotting signal bands is analyzed by software Image J **(F)**. (n=2, *P<0.05; **P<0.01; ***P<0.001; ****P<0.0001).

Moreover, we have also evaluated the effects of RT activity on BmCPV infection. Our results indicated that expression levels of vp1 gene and VP7 protein in BmCPV-infected cells pretreated with AZT increased, while the results were the opposite when cells were pretreated with GSK-LSD1 (**Figures 6A–C**). Moreover, we have also found that treatment of cells with GSK-LSD1 could enhance the ability of vcDNA-S7 to inhibit BmCPV infection (**Figures 6D–F**). These results support the notion that RT activity cooperates with vcDNA-S7’s antiviral function.

### vcDNA-S7 Provides vsiRNAs for RNAi Pathway

**Figure 7:**
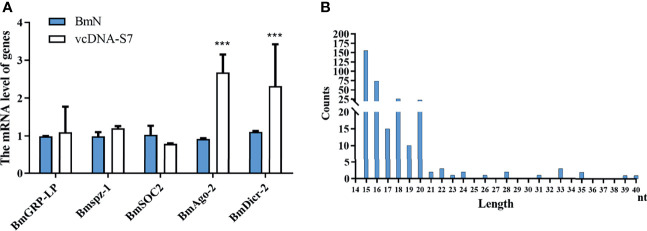
vcDNA-S7 can provide vsiRNAs for RNAi pathway. **(A)** Effects of vcDNA-S7 on the main immune pathways of *mori*. BmN cells (1×10^6^) were transfected with 1μg vcDNA-S7 for 48 h. The relative RNA level of the key genes in the immune pathways (including RNAi, JAK-STAT, Imd, and Toll pathway) was determined by real-time PCR (n=2, ***P<0.001). **(B)** 1×10^7^ BmN cells were transfected with 6 μg vcDNA-S7 for 48 h and small RNAs were deep sequenced. Size distribution of vsiRNAs derived from vcDNA-S7 was shown.

To understand the antiviral mechanism of vcDNA-S7, we tested the effects of vcDNA-S7 on key genes related to the innate immune pathway (including RNAi pathway, JAK-STAT pathway, Imd pathway, and Toll pathway) in silkworm. In BmN cells transfected with vcDNA-S7, the expression levels of peptidoglycan-recognition protein LB (*BmPGRP-LB*, Imd pathway), suppressor of cytokine signaling 6 (*Bmspz-1*, JAK-STAT pathway), and *BmCS2* (Toll pathway) were not significantly changed, whereas the expression levels of *BmDicer-2* and *BmAgo-2* (RNAi pathway) were upregulated (**Figure 7A**), indicating that RNAi pathway was activated by vcDNA-S7. Since the molecules examined by the RNAi system are heterologous dsRNA while vcDNA-S7 is a DNA molecule, how is RNAi pathway activated by vcDNA-S7. Previous studies have indicated that RNA virus-derived circular DNAs can serve as a template for the biogenesis of antiviral siRNAs (33). To determine if vcDNA-S7 can serve as a template for generation of virus-specific siRNAs, we transfected BmN cells with vcDNA-S7, and the purified small RNAs at 48 h post transfection were subjected to deep sequencing. Using vcDNA-S7 as a reference sequence, we detected a total of 200 vcDNA-S7-specific viral small RNAs within a range of 15 to 40 nt (**Figure 7B**). The predominant classes of viral small RNAs are 15-nt and 16-nt. Besides, an abundant 20-nt vsiRNAs class originating from vcDNA-S7 was also observed, which is considered to be the product of Dicer-2 (25). These data suggest that vcDNA-S7 may serve as a template for the biogenesis of antiviral siRNAs.

## Discussion

In the present study, we have shown that a circular DNA vcDNA-S7 derived from BmCPV belonging to double-stranded RNA virus can be generated in the infected cells by endogenous RT and vcDNA-S7 can serve as a template for the biogenesis of antiviral siRNAs. Active RTs can be encoded by a series of intracellular retroelements, including long terminal repeat (LTR) retrotransposons, non-LTR retrotransposons, and endogenous retroviruses (34). Although most retroelements have been mutated during evolution, their open reading frames encoding RT remain mostly intact. In *Drosophila* and mosquito, LTR retrotransposons have been reported to be the main source of the reverse-transcriptase activity (8, 9, 35). In mouse embryonic stem cells, retroelements encoding MusD appear to provide a RT activity (32). In this study, silkworm RTs were clustered into 9 groups based on the sequence similarity, and two groups of them (including 18 genes) were found to be involved in the formation of vcDNA-S7. Domain analysis has indicated there are typical exonuclease-endonuclease phosphatase and RT superfamily domains in RT of these two groups, implying these two distinct domains might be critical for the formation of the vcDNA-S7. It has been previously reported that, in cells infected by virus, such as DCV, FHV, SNV, and CHIKV, viral genomic RNA forms chimeric DNA of virus-derived DNA as well as LTR-retrotransposon mediated by LTR-retrotransposon (8, 9). In this case, LTR sequences can mediate these chimeras to form chimeric circular DNAs by DNA repair mechanisms (8, 9). However, in this study, we found that vcDNA-S7 is completely derived from BmCPV genomic dsRNA S7 and does not contain any LTR sequence. Thus, it is unlikely that vcDNA-S7 could be formed by homologous end repair mediated by LTR sequence, thus suggesting that the mechanism of vcDNA-S7 formation may be distinct from previous studies (8, 9). In fact, previous reports have suggested that FHV may form a DNA molecule entirely derived from truncated genome of FHV but, nevertheless, unrelated to chimeras of viral DNA and LTR sequence (8). Moreover, it has been reported that a DNA complementary to vesicular stomatitis virus (VSV) (a non-retroviral RNA virus) RNA can be generated by reverse transcription *via* the long-interspersed element-1 retrotransposons (36). Interestingly, only virus-derived DNA sequences were detected, but no host-virus chimeric DNA sequences were found. Based on this observation, the authors proposed that VSV RNA alone is reverse transcribed into DNAs and that the virus-derived DNAs are present as extrachromosomal DNAs (36). However, the details of reverse transcription mechanism are unknown in this model. The route of vcDNA-S7 formation may be similar to that of VSV. Moreover, it is not yet known whether the formation of vcDNA-S7 does depend on the sequences flanking the split sites of vcDNA-S7. Further mechanistic studies focusing the formation mechanism of vcDNA-S7 are still warranted.

In the present study, we found that vcDNA-S7 is able to diminish BmCPV gene expression both *in vivo* and *in vitro*, implying that BmCPV-derived vcDNA-S7 produced by silkworms plays an important role in controlling the process of viral infection. Important antiviral immune cascades present in silkworm include RNAi, JAK-STAT, IMD, and Toll signaling pathways (13). In this study, vcDNA-S7 was found to activate the RNAi pathway, but not other antiviral pathways. Our previous study also showed that BmCPV infection is unable to activate silkworm JAK-STAT, IMD, and Toll antiviral pathways, mainly activating the RNAi pathway (13). It has confirmed that FHV-derived vcircDNAs can be stably and continuously transcribed, thus elevating dsRNA yields. This increase on dsRNA levels leads to a higher abundance of viral vsiRNAs in *Drosophila*, which may boost an RNAi-mediated antiviral immune response (8, 9, 35). In this study, Small RNA deep sequencing showed that vcDNA-S7 mediated by RT can serve as a template for the biogenesis of antiviral siRNAs. To date, the study have demonstrated that, in silkworms, the predominant vsiRNA class generated by Dicer-2 is 20-nt in length (25). Herein, we also observed an abundant 20-nt vsiRNAs class originating from vcDNA-S7, which is considered to be a Dicer-2 product. However, another predominant classes of vsiRNAs were those of 15-nt and 16-nt. Previous study showed that an abundant 17-nt vsiRNAs class originating from BmCPV S7 segment was observed, which are produced by a yet-to-be-defined mechanism (25). Therefore, we guessed that the predominant classes of 15-nt, 16-nt vsiRNAs are also produced by an unknown RNase of silkworm. Generally, it is thought that Dicer-2 acts on the cleavage of BmCPV dsRNAs into vsiRNAs (particularly on the silkworm surveillance of BmCPV genomic dsRNAs), and then act on viral RNAs to defend against antiviral infection (25). Our study provides a novel explanation for the source of vsiRNAs in *B. mori* resistance to BmCPV by mediating RNAi pathway. Studies have also shown that vcircDNAs are formed during the process of recognizing self vs. non-self in mosquito (37). Thus, a response of host towards the virus genome to produce vcircDNAs can be regarded as a defense against virus. In *Drosophila*, vcircDNAs derived from FHV can improve the tolerance of *Drosophila* to FHV (9). *B. mori* cytoplasmic polyhedrosis caused by BmCPV infection is a chronic disease, which may also be related to the presence of circular DNAs derived from BmCPV.

One latest study has shown that the endogenous reverse-transcriptase can convert the viral RNA into DNA in mouse embryonic stem cells during virus infection. Thereafter, these viral DNAs and viral RNAs form a DNA/RNA hybrid structure. This DNA : RNA heteroduplex recruits RNase H1 to hydrolyze the viral RNA present in this hybrid, resulting in the suppression of viral replication (32). Our preliminary results showed that knockdown of silkworm RNase H1 promote BmCPV gene expression. Conversely, overexpression of RNase H1 inhibits BmCPV gene expression (data not shown), suggesting that RNase H1 plays an important role in BmCPV infection. RNase H proteins are the most ancient and abundant proteins in eukaryotes. In this context, the production of vcDNA by endogenous RT and the RNase H1-mediated antiviral pathway could also be involved in the control of virus infection in insects. Further mechanistic studies resolving this topic should be performed in the future.

To combat viral infection, multicellular organisms evolved mechanisms to limit replication of viral pathogens. Adaptive immunity (also known as specific immunity) is an effective means to combat viral infection, where the organism obtains immune memory due to pathogen infection to resist any re-infection process eventually conducted the pathogenic agent. It is well known that, in jawed vertebrates, a protein-based antiviral response provides a reservoir of immunological memory to target specific viral pathogens. Immunological memory also exists in nucleic acid immunity. For instance, CRISPR-Cas9 is related to an adaptive immune defense, developed by bacteria and archaea during long-term evolution, against invading viruses and foreign DNA (38). vcDNA-mediated RNAi antiviral response is essentially one subtype of nucleic acid immunity. Recent studies have shown that RNAi antiviral effects, mediated by RNA virus-derived vcircDNAs, can be transferred in insects, thus allowing offspring to possess immune memory and be protected from invasion by the same pathogen (39). In this study, vcDNA-S7 can also be found in other tissues, including gonads in addition to the midgut, suggesting that the generated vcDNA-S7 in BmCPV-infected midgut can be transferred into other tissues in some other manners. According to our study, vcDNA-S7 may exist as episomes (a extrachromosomal genetic determinant which can reproduce autonomously or as an integral part of the chromosomes) (37) or integrated into silkworm genome to create a DNA-based template for an antiviral response appears to allow some sustained immunological memory.

In addition to its role in antiviral response, circular DNAs are commonly found in a number of species, including *Drosophila* (40), Arabidopsis (41), yeast (4), mouse (5), and human cell lines (42). Circular DNAs have attracted increasing attention due to their various biological functions, including driving genome rearrangement (43), carrying enhancers to regulate the expression of certain oncogenes (44), accelerating adaptive evolution (6), and stimulating innate immunity (45). In this regard, vcDNA-S7 may have similar biological functions which need to be further explored.

**Figure 8:**
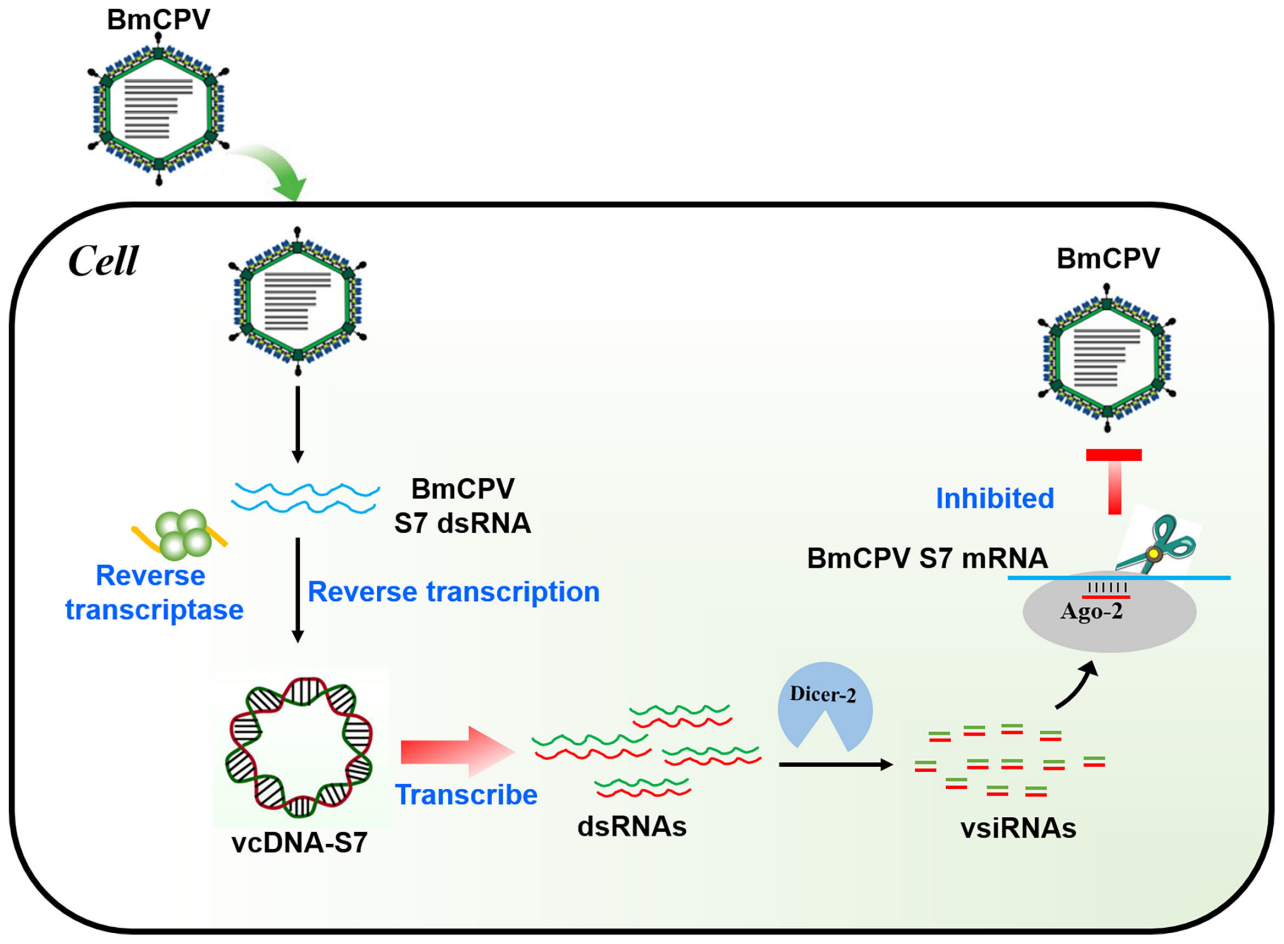
Mechanism of BmCPV-derived circular DNA vcDNA-S7 controlling BmCPV infection. Silkworm infected with BmCPV produce viral-derived vcDNA-S7 mediated by reverse transcriptase. vcDNA-S7 can be transcribed to RNA and produce vsiRNAs to control BmCPV infection by RNAi pathway.

In conclusion, BmCPV can produce a viral-derived vcDNA-S7 mediated by endogenous RT and vcDNA-S7 can serve as a template for the biogenesis of antiviral siRNAs, resulting in the repression of BmCPV infection (**Figure 8**). To our knowledge, this is the first report disclosing the formation of vcircDNAs by double-stranded RNA viruses. This research not only provides a new perspective to understand the genetic information carried by BmCPV genome, but also provides new directions to study the interplay between BmCPV and their hosts.

## Data Availability Statement

The datasets presented in this study can be found in online repositories. The names of the repository/repositories and accession number(s) can be found below: https://www.ncbi.nlm.nih.gov/, SRR16977180 https://www.ncbi.nlm.nih.gov/, SRR17050376.

## Author Contributions

CG was responsible for conception and design of the study. MZ and JP were responsible for data acquisition and analysis and all other authors were responsible for drafting manuscript and figures. RX, GC, and XH were responsible for providing general guidance. All authors contributed to the article and approved the submitted version.

## Funding

This study was funded by the National Natural Science Foundation of China (32072792, 31972620 and 31872424), China Postdoctoral Science Foundation (2019M661937, 2019M651952) and Priority Academic Program of Development of Jiangsu Higher Education Institutions.

## Conflict of Interest

The authors declare that the research was conducted in the absence of any commercial or financial relationships that could be construed as a potential conflict of interest.

## Publisher’s Note

All claims expressed in this article are solely those of the authors and do not necessarily represent those of their affiliated organizations, or those of the publisher, the editors and the reviewers. Any product that may be evaluated in this article, or claim that may be made by its manufacturer, is not guaranteed or endorsed by the publisher.
