# Supplementary material for: BmCPV-Derived Circular DNA vcDNA-S7 Mediated by Bombyx mori Reverse Transcriptase (RT) Regulates BmCPV Infection
Source: Front Immunol. 2022 Mar 15;13:861007. doi: 10.3389/fimmu.2022.861007 (PMC8964962; doi:10.3389/fimmu.2022.861007)
Supplement: Supplementary file 1 [file DataSheet_1.pdf]

Table S1. Primers sequences used in this study.

Gene name	Forward (5'➞3')	Reverse (5'➞3')
XM_038020758.1 123(Group 1)	AAGGCTTGTGCCAAAGGTCG	GAACGAGGATCCGTCGGAAG
XM_038021438.1-126(Group2)	GTCTCCAGAGCCACGTCAAA	TCTGTTGGAGAACCGTGTCG
FJ265562.1-2511(Group 3)	GATCGACCAAAACCCTCCAGA	GACCTCAGAGAGGCCGACTA
GU815090.1-123(Group 4)	AGCATAAGGTGGTTGGTCCG	CGCTGTGGAGCCCTTCTTAT
FJ265554.1-159(Group 5)	CGGGCTCGGCTAATGAAGTT	GGCTTTTGCACCAGTCCATC
U07847.1-13(Group 6)	AAACTTGCATAAGGCCCGTC	CGCTGACTTCATGTATTTCCGA
XM_038017410.1-32(Group 7)	AGACTTAAGGATGCCAACTGGT	GATGGAGGAATATTACTGTCCCA
XM_038010567.1-1108(Group 8)	ATTTTGTTGGGTGCCAGGTC	AGAGTAAACTTGAAACCTTTCGTT
LOC110384916-227(Group 9)	GTTACCGCAGCTAACAAGCG	ACGATGGGCGAACTTGTGAT
BmGRP-LP	CACTGCAACAGAAAGCTGTAG	CGCAATATGCCGATCCGTCAC
Bmspz-1	GAGCGTTATGGACGAGAAGC	CCTGGTTGCCGTGGACTATG
BmSOCS2	GTGACAGACCGTTGGCTAGG	GCACCGGCGAGTGTGGACAC
BmAgo-2	GAACTGACTGGATTGCATCGC	GGGCAGGAAGAATCTCGTGT
BmDicer-2	AGTACCCCTGGGATCAACGA	CGATATCGGAGCCGACACAA
Bm CPV vp1	GGTCTCGACGTGAATACCGA	TCGTCTGCTTCACTAGCACG
TIF-4A	GAATGGACCCTGGGACACTT	CTGACTGGGCTTGAGCGATA
vcDNA-S7	GCAGGCAGAACCGCA	AGTTCACGCCAATGT

Table S2. The siRNA sequences used in this study.

Target genes	Sense (5'➞3')	Anti-sense (5'➞3')
XM_038020758.1 123(Group 1)	CCAAUAUGGUUUCCGGAGATT	UCUCCGGAAACCAUAUUGGTT
XM_038021438.1-126(Group 2)	GGUCCUAUUACACCGCCUUTT	AAGGCGGUGUAAUAGGACCTT

FJ265562.1-2511(Group 3)	GCUACCCACACUCUACAAATT	UUUGUAGAGUGUGGGUAGCTT
GU815090.1-123(Group 4)	GAGUAGCAUGUUUGAUGUGUUTT	AACACAUCAAACAUGCUACUCTT
FJ265554.1-159(Group 5)	CCGCUUCGCUACAUUGAAUTT	AUUCAAUGUAGCGAAGCGGTT
U07847.1-13(Group 6)	GUCUGGCACAAUGGUUUGAUUTT	AAUCAAACCAUUGUGCCAGACTT
XM_038017410.1-32(Group 7)	GUCCCUUACUUUACAAUAUTT	AUAUUGUAAAGUAAGGGACTT
XM_038010567.1-1108(Group 8)	GCAACAAUCCGAAGCCAUUTT	AAUGGCUUCGGAUUGUUGCTT
LOC110384916-227(Group 9)	GCUCAGAUCCGUCCAACUATT	UAGUUGGACGGAUCUGAGCTT

Figure S1

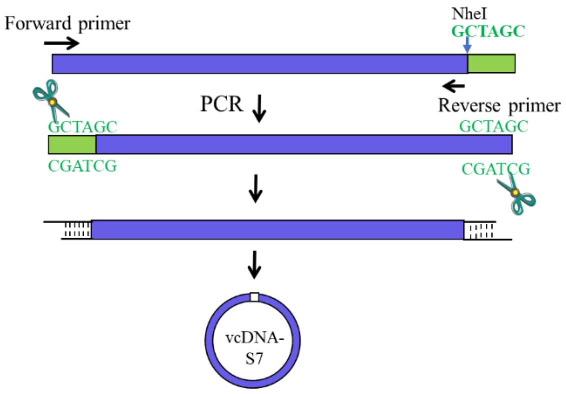

Figure S1. The flowchart for the in vitro synthesis of vcDNA-S7. The specific primers carrying an NheI site were designed and then PCR was performed using a plasmid which contained the full-length cDNA of BmCPV S7 segment as a template (pIZT-CS7). The amplified product was further digested with NheI and then circularized to obtain vcDNA-S7, using T4 DNA ligase.
